# Comparing Ocular Toxicity of Legacy and Alternative Per- and Polyfluoroalkyl Substances in Zebrafish Larvae

**DOI:** 10.3390/toxics11121021

**Published:** 2023-12-14

**Authors:** Han-seul Lee, Soogyeong Jang, Youngsub Eom, Ki-Tae Kim

**Affiliations:** 1 Department of Environmental Engineering, Seoul National University of Science and Technology, Seoul 01811, Republic of Korea; 2 Zebrafish Translational Medical Research Center, Korea University, Ansan 15355, Republic of Korea; 3 Department of Ophthalmology, Korea University Ansan Hospital, Ansan 15355, Republic of Korea; 4 Department of Ophthalmology, Korea University College of Medicine, Seoul 02841, Republic of Korea; 5 Department of Environmental Energy Engineering, Seoul National University of Science and Technology, Seoul 01811, Republic of Korea

**Keywords:** eye toxicity, neurotoxicity, PFOS, PFOA, PFBS, PFBA

## Abstract

Studies comparing the ocular toxicity potential between legacy and alternative PFAS are lacking. To address this research gap, zebrafish larvae were exposed to both legacy PFAS (i.e., perfluorooctanesulfonic acid [PFOS] and perfluorooctanoic acid [PFOA]) and their corresponding alternatives (i.e., perfluorobutanesulfonic acid [PFBS] and perfluorobutanoic acid [PFBA]). Alterations in their visual behaviors, such as phototactic and optomotor responses (OMR), were assessed at sublethal concentrations. Gene expression variations in visual function-associated pathways were also measured. Visual behavioral assessment revealed that PFOS exposure resulted in concentration-dependent reductions in phototactic responses at 10–1000 μg/L, with PFOA exerting reduction effects only at 100 mg/L. However, their two alternatives had no effect at all tested concentrations. Following an improved contrast-OMR (C-OMR) assessment, PFOS decreased the OMR to a water flow stimulus at 10, 100, and 1000 μg/L. The gene expression analysis revealed that PFOS exposure markedly downregulated most genes involved in the opsins in the photoreceptor and phototransduction cascade, which explains the observed visual behavior changes well. Our findings indicate that PFOS is the most likely PFAS to cause visual toxicity, with PFOA present but less likely, and their substitutes, PFBS and PFBA, cannot be classified as visually toxic to zebrafish.

## 1. Introduction

Per- and polyfluoroalkyl substances (PFAS) are mass-produced industrial chemicals widely used in everyday consumer products, such as carpets, emulsion stabilizers, and skin conditioners. However, due to environmental persistence and bioaccumulation in the food web, the EU persistent organic pollutant regulation has prohibited the use and production of long-chained PFAS, such as perfluorooctanesulfonic acid (PFOS) and perfluorooctanoic acid (PFOA), since 2009 and 2020, respectively [1]. Despite efforts on phasing out long-chained PFAS, the so-called legacy PFAS, these substances remain predominant in human samples and the environment. For example, while the serum PFOA and PFOS concentrations have increased in certain regions of China [2], monitored concentrations could reach levels of 70 ng/L of PFOA and PFOS in public water systems in the USA [3]. 

Regulations on legacy PFAS and voluntary exits of companies are driving the emergence and increasing production of short-chained PFAS (also known as alternative PFAS). Accordingly, perfluorobutanesulfonic acid (PFBS) and perfluorobutanoic acid (PFBA) have become representative alternatives to PFOS and PFOA, respectively. Alternative PFAS tend to exhibit concentrations similar to legacy PFAS in human milk samples [4]. Concentrations of alternative PFAS were found to exceed those of legacy PFAS in the serum of residents of an industrial park located in China. PFBS and PFBA also exhibited peaked concentrations of 3.78 and 3.70 μg/L in river samples, exceeding those of PFOA 1.95 μg/L [5]. The increasing concentration and frequent detection of alternative PFAS have raised concerns about their potential negative effects [6], but studies have reported that the short-chained alternative PFAS may exhibit lower persistence and bioaccumulation than legacy PFAS [7]. More studies are required to provide deeper insights into the safety of alternative PFAS compared to legacy PFAS. 

For most fish species, visual system impairment is a significant threat to survival because it affects relevant survival factors, such as orientation, schooling, predator avoidance, prey capture, and reproduction [8]. Because visual behavior alterations can result in population decline and serious ecological impacts due to reduced individual physical conditions [9], previous studies have evaluated the ocular toxicity of teleost fish following exposure to diverse chemicals such as dioxin-like pollutants and metals [10,11,12,13]. However, studies exploring PFAS-induced ocular toxicity are few. Chen et al. (2018) reported that PFBS accumulation in the eyes induced an impaired visual system in marine medaka [6]. Wu et al. (2022) reported disrupted eye development and changes in locomotor behaviors in zebrafish caused by F53B, another alternative to PFOS [14]. Nevertheless, these studies focused on ocular toxicity concerning malformations in eye embryogenesis and changes in locomotor behavior, which are not closely related to the effects of visual dysfunction-oriented behavior. Furthermore, a systematic study is needed to compare the ocular toxicity between legacy and alternative PFAS within a single toxicity testing system to ensure the safety of alternative PFAS. 

This study aimed to evaluate the ocular toxicity between two legacy PFAS (i.e., PFOS and PFOA) and two corresponding short-chained alternatives (i.e., PFBS and PFBA) in zebrafish embryos. Developmental toxicity testing was first conducted to determine the sublethal concentrations of each PFAS and to assess phototactic responses in zebrafish larvae. The optomotor response (OMR) was modified to enhance the contrast sensitivity to responses to a repeating pattern, and this developed contrast-OMR (C-OMR) was then employed to evaluate OMR changes. In addition, changes in the expression of visual system-associated genes were measured to investigate the underlying ocular toxicity mechanism.

## 2. Materials and Methods

### 2.1. Chemicals

PFOS (CAS No. 1763-23-1, purity > 98%), PFOA (CAS No. 335-67-1, purity > 95%), PFBS (CAS No. 375-73-5, purity > 98%), PFBA (CAS No. 375-22-4, purity > 98%), and dimethyl sulfoxide (DMSO) were purchased from Sigma–Aldrich (St. Louis, MO, USA). 

### 2.2. Embryo Collection and Developmental Toxicity Testing

Zebrafish (*Danio rerio*, AB-wild type) were cultured in a continuous flow-through system (Tecniplast, Buguggiate, Italy). Following zebrafish breeding protocols [15], fish were kept in a stable water temperature of 28 ± 1.0 °C and a cycle of 14 h light and 10 h dark conditions. The system water was filtered by reverse osmosis, and the pH of 7.0–7.4 and conductivity of 450–500 μS/cm were maintained using sea salt (Instant Ocean^®^, Blacksburg, VA, USA). Commercial dried food (Gemma Micro 300, Skretting, Fukuoka, Japan) was fed 5 mg per fish three times daily for six days a week. Zebrafish embryos were obtained from spawning adults in a water bath overnight, and spawning was induced in the morning when the lights were turned on. Healthy embryos, free of coagulation and bubbles, were selected during developmental stages 2–4 h post fertilization (hpf) for further experiments under a stereomicroscope. 

Zebrafish embryos were exposed to PFOS, PFOA, PFBS, and PFBA at 1, 10, 100, and 1000 μg/L. These concentrations were determined to cover environmentally relevant concentrations detected in water. For example, the highest detected concentration of PFOA in surface water samples was 11 μg/L in Alabama, USA [16], and PFBS was detected in the leachate of a landfill site in Singapore at 1.92 μg/L [17]. For developmental toxicity testing, 96 embryos (32 per replicate), randomly selected, were used for each PFAS concentration. Embryos at 2–4 hpf were placed in a sterile 96-well plate with 100 μL of E2 culture medium containing the test solution per well and exposed to the desired PFAS concentration. At 5 days post-fertilization (dpf), the embryonic lethality and malformation were evaluated under a stereomicroscope. The screened malformations were pericardial edema, yolk sac edema, hemorrhaging, and bent spine. 

### 2.3. Phototactic Response Assay

Phototactic response assays were conducted at the determined sublethal concentrations of 1, 10, 100, and 1000 μg/L for each PFAS based on embryonic mortality and malformations. For visual behavior tests, 25 embryos at 2–4 hpf were randomly selected per well in a sterile 6-well plate, with three replicates per concentration. At 5 dpf, malformed or dead larvae were excluded from the assessment. The plates were covered with parafilm to prevent evaporation. Phototactic response assessment was conducted as described by Brockerhoff et al. (1995) [18]. A black acrylic box measuring 10.5 × 3 × 4 cm (length × width × height) was designed, which comprised two chambers separated by an intermediate sliding partition. The left chamber was open for the light condition, and the other was covered for the dark condition. To monitor the dark-to-light transition stimulation of larvae between the unexposed and PFAS-exposed groups, all larvae were first placed in the right chamber and acclimated to the dark condition for 5 min. The partition was then removed, and the uncovered left chamber was illuminated under ambient light (200 lx), while the light chamber was covered to keep it dark. After 1 min, the compartment was returned into the box, and the number of remaining larvae in the left chamber was counted.

### 2.4. C-OMR (Contrast-Optomotor) Assay

C-OMR analysis was performed to evaluate the visual development and visual motor function in zebrafish larvae after PFOS exposure at 10, 100, and 1000 μg/L. The C-OMR analysis was performed only with PFOS, because only PFOS, among the four FPASs tested, caused changes in the phototactic response behavior at concentrations from 10 to 1000 μg/L. This concentration falls in the 0.5–40 μg/L in Germany [19] and 0.03–22.6 µg/L in China [20,21,22], which is the level detected in human blood. The newly developed methodology for C-OMR assessment was employed following the research of Kwon et al. (2021) [23]. The existing contrast sensitivity test, the so-called OMR assay, was improved to more efficiently explore the visual function of the zebrafish larvae by providing various gray color gradients. A total of 16 levels were devised from G1 for black to G16 for white. In this study, 8 gradients (i.e., G4, G7–G13) were given. As shown in Figure S5, a band of grades G4 flowing to the left was used to move the zebrafish larvae to the starting point, and then white and gray bands comprising G7–G13 in the opposite direction were applied sequentially to move the zebrafish larvae to the end point. In the order of G4 ← G7 → G4 ← G8 → G4 ← G9 → G4 ← G10 → G4 ← G11 → G4 ← G12 → G4 ← G13 →, the images comprising the eye stimulation preparation section (G4) and assessment section (G7–13) were regenerated for 30 s at a 1 s interval, respectively. The bands were designed to pass in one direction at a rate of 2.6 cycles/s for 30 s, and all conditions were optimized in the preliminary experiments. The produced video was played on a tablet device (Galaxy Tab 10.1, Samsung Electronics Co. Ltd., Seoul, Republic of Korea) under a clear acrylic tray comprising six lanes. The movements of the larvae according to the contrast sensitivity grade used were recorded using an image-recording device (FDR-AX700, Sony, Tokyo, Japan). 

For C-OMR analysis, 25 embryos at 2–4 hpf were randomly selected in a sterile 6-well plate per well, with three replicates per concentration. At 120 hpf, malformed or dead larvae were excluded from the analysis. Prior to the C-OMR analysis, the 120 hpf zebrafish larvae per concentration were acclimated in the dark for 40 min and received one training session with video images identical to the experiment. The C-OMR analysis results were expressed by calculating the ratio of larvae located at the starting point over time, as the area under the curve (AUC) for each contrast sensitivity grade. Smaller values of AUC indicate a faster response to stripe stimuli, and higher values indicate a lower response or low sensitivity to stimuli. For the preliminary experiment, the methodologies on the AUC ratio of the larvae located at the origin and each contrast sensitivity grade section were validated under exposure to gentamicin, a positive chemical. 

### 2.5. Gene Expression Measurement

To understand the outcomes of behavioral screens for visual system damage, we identified and selected 14 genes related to photoreceptors and the phototransduction cascade within the retina, where an impairment of related genes was observed in visual mutants. Subsequently, the expression patterns of these selected genes were quantitatively analyzed after exposing zebrafish embryos to PFOS at 10, 100, and 1000 μg/L for 5 days. The analyzed genes related to photoreceptors included cone-rod homeobox (*crx*), rhodopsin (*rho*), opsin 1 short-wave-sensitive *2* (*opsn1sw2*), opsin 1 medium-wave-sensitive 1 (*opn1mw1*), and opsin 1 long-wave-sensitive 1 (*opn1lw1*) [24]. The analyzed genes related to phototransduction included recoverin 3 (*rcvrn3*); cyclic nucleotide gated channel subunit alpha 1b (*cnga1b*); guanylate cyclase activator 1d (*guca1d*); guanylate cyclase activator 1e (*guca1e*); guanylate cyclase 2D, retinal (*gucy2d*); arrestin 3a, retinal (X-arrestin) (*arr3a*); arrestin *3b*, retinal (X-arrestin) (*arr3b*); phosphodiesterase 6H, cGMP-specific, cone, gamma, paralog a (*pde6ha*); and G protein subunit alpha transducin 2 (*gnat2*) [24,25,26].

RNAs were extracted following the manufacturer’s protocol using the AllPreP Fast DNA/RNA Mini kit (Qiagen, Hilden, Germany). Total RNA was extracted from three replicates of 15 larvae for each PFOS concentration. Following extraction, the total concentration and quality of RNA were estimated using a spectrophotometer (Thermo Fisher Scientific, Waltham, MA, USA), after which the RNA samples were stored at −80 °C. A High-Capacity cDNA Reverse Transcription kit (Applied Biosystems, Waltham, MA, USA) was utilized to decontaminate genomic DNA from total RNA by synthesizing complementary DNA via RT-PCR and gene transcription patterns. 

The primer sequence for the target gene was designed with the NCBI Primer Blast, and the most suitable primers were selected. Their sequences are listed in Table S1. The targeted 14 visual system-related genes, categorized as photoreceptors and the phototransduction cascade, were analyzed with 3 replicates each. Beta-actin, a commonly used housekeeping gene in the zebrafish gene expression analysis, was selected as a reference gene due to its stability and high expression levels in all tissues under various conditions, providing a reliable and accurate method of normalization and reducing errors in the gene expression analysis [27]. The qRT-PCR analysis was performed using Faststart DNA Master SYBR Green. For the reaction, SYBR green 10 μL, H_2_O 6.8 μL, forward primer 0.6 μL, reverse primer 0.6 μL, and 2 μL of cDNA diluted to 5 ng/μL were mixed. Using a LightCycler 96 (Roche, Basel, Switzerland), we set the cycling parameters to 95 °C for 10 min, followed by 45 cycles of 95 °C for 10 s, 55–60 °C for 10 s, and 75 °C for 10 s. Then, we amplify it with a melting sequence of 95 °C for 10 s, 65 °C for 60 s, and 97 °C for 1 s to obtain Ct values. The obtained Ct value was applied to the 2^−ΔΔCt^ method [28], and the expression level of each target gene was analyzed as the mRNA amount of the housekeeping gene. 

### 2.6. Statistical Analysis

The results were analyzed using SigmaPlot software 13 (Systat Software Inc., San Jose, CA, USA) and presented as mean ± SEM (standard error of the mean). For the developmental toxicity test and quantification of gene expression, the statistical significance was assessed using one-way ANOVA, followed by Dunnett’s post hoc test. Regarding the phototactic response and C-OMR assay, the significance was determined using the one-way ANOVA and t-test, followed by Dunnett’s post hoc test. Significant differences were denoted as *p* < 0.05 (*), *p* < 0.01 (**), and *p* < 0.001 (***). The normality and equality of variance were evaluated using the Shapiro–Wilk and Brown–Forsythe tests, respectively.

## 3. Results and Discussion

### 3.1. Developmental Toxicity after PFOS, PFOA, PFBS, and PFBA Exposure

Embryonic mortality and malformations were evaluated after exposure to PFOS, PFOA, PFBS, and PFBA at 1, 10, 100, and 1000 μg/L. As shown in Figure S1, at all concentrations of the four PFAS, the incidence of mortality and malformations was not significant compared to the control, as it was less than 10%, which satisfies the OECD TG236 (Fish Embryo Acute Toxicity Test; OECD, 2013) [29]. Owing to low developmental toxicity, the same concentration ranges were applied for further visual behavioral screens. 

### 3.2. Changes in Phototactic Responses after PFOS, PFOA, PFBS, and PFBA Exposure

For the first screening of ocular toxicity of PFAS, changes in the phototactic response of zebrafish larvae were measured after the exposure to four PFAS at 1, 10, 100, and 1000 μg/L. The adequacy of the phototactic response assessment was verified using gentamicin, a substance known to induce ocular burning and irritation in humans. Gentamicin has been commonly utilized as a positive control in visual behavior assays, such as the optokinetic response (OKR) and visual motor response in zebrafish models [30]. As expected, the exposure to 30 μM gentamicin had a significant inhibitory effect on larval behavior in response to light stimuli (Figure S2). The observed abnormal behaviors in zebrafish larvae exposed to gentamicin corroborated previously reported findings [20], confirming the suitability of the methodology of OMR employed in this study. 

**Figure 1:**
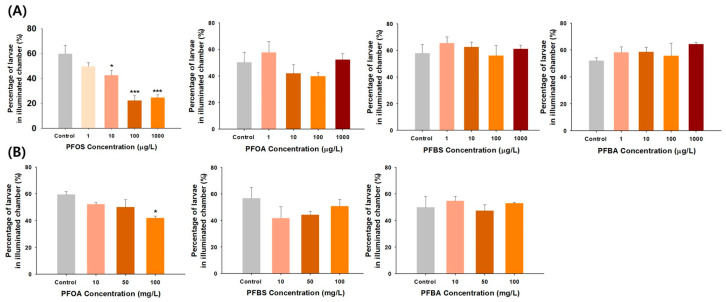
(**A**) Phototactic response of zebrafish larvae exposed to PFOS, PFOA, PFBS, and PFBA at concentrations of 1, 10, 100, and 1000 μg/L. (**B**) Phototactic response of zebrafish larvae exposed to PFOA, PFBS, and PFBA at 10, 50, and 100 mg/L. Statistical significance was denoted by * *p* < 0.05, *** *p* < 0.001.

Figure 1 presents the phototactic response results in zebrafish larvae exposed to the four PFAS. The PFOS-treated group exhibited a concentration-dependently marked decrease in the proportion of larvae moving from the dark chamber to the illuminated chamber at 10, 100, and 1000 μg/L. However, exposure to PFOA, PFBS, and PFBA at the tested concentrations induced no notable inhibition of phototactic response in zebrafish larvae. Therefore, the phototactic response tests were repeated by increasing the PFOA, PFBS, and PFBA concentrations to 10, 50, and 100 mg/L. As illustrated in Figure 1, a decrease was observed in the PFOA-treated group, with a statistically significant difference occurring only at 100 mg/L. In contrast, no changes in phototactic response were observed in the PFBS- and PFBA-treated groups despite the concentration increases. Larvae with intact visual function tend to exhibit positive phototactic behavior, such as escaping from darkness to light, to increase the chances of avoiding predators and finding food to survive and thrive [31]. The absence of this positive phototactic response indicates an impaired vision system [32]. Collectively, legacy PFOS and PFOA are toxicants capable of impeding a positive phototactic response due to vision impairment in zebrafish larvae, resulting in phototactic behavior changes. In contrast, alternatives (i.e., PFBS and PFBA) appear to be relatively safe regarding phototactic behavior effects. To our knowledge, this study is the first to show that PFOS is more ocular toxic than PFOA. Previous studies have also reported a higher toxicity of PFOS than PFOA in terms of zebrafish embryonic toxicity and neurobehavior alterations [33,34].

This is the first study to investigate the effects of PFAS on phototactic behaviors employed to monitor the ability to swim toward illuminated regions. Fernandes et al. (2012) showed that brain photoreceptors regulate locomotor responses to light in zebrafish larvae by mediating it through sensory neurons [35]. Therefore, PFAS, particularly PFOS, is thought to inhibit the development of photoreceptor and visual function during embryogenesis, resulting in an impaired phototactic response. As the phototactic response in larvae is variable and insufficient to serve as an independent screening method [18], previous studies have combined two visual behavior assays, such as the phototactic response and OKR, or OKR and OMR, to screen for visual system defects in zebrafish larvae [18,36].

### 3.3. PFOS-Induced Changes in C-OMR Behavior

To more accurately assess the PFOS-induced visual dysfunction in zebrafish, we performed a second round of visual behavior screening, known as the C-OMR, at 10, 100, and 1000 μg/L. Similar to the phototactic assessment, we first investigated visual behavior alterations in the employed C-OMR under exposure to 30 μM gentamicin, as a positive control. As shown in Figure S3, in the untreated negative and gentamicin-treated positive controls, the number of larvae at the starting point decreased over time in all contrasts for each contrast sensitivity grade (G7–G13), indicating that the zebrafish responded appropriately to the band flow stimulus. We also observed a trend for slower movement from starting to end point in the gentamicin-treated group, as the contrast sensitivity decreased compared to the negative control. In C-OMR experiments, swimming inhibition, a reduced percentage of larvae in the starting point curve over time, can be determined based on the AUC area. As the speed of reaching the destination slowed, the AUC area showed a large value. As shown in Figure S4, the exposure to gentamicin caused a significant increase in the converted AUC area in all bands except grade G8. The increased AUC values observed as the contrast sensitivity grade decreased confirmed the utility of the C-OMR assay.

**Figure 2:**
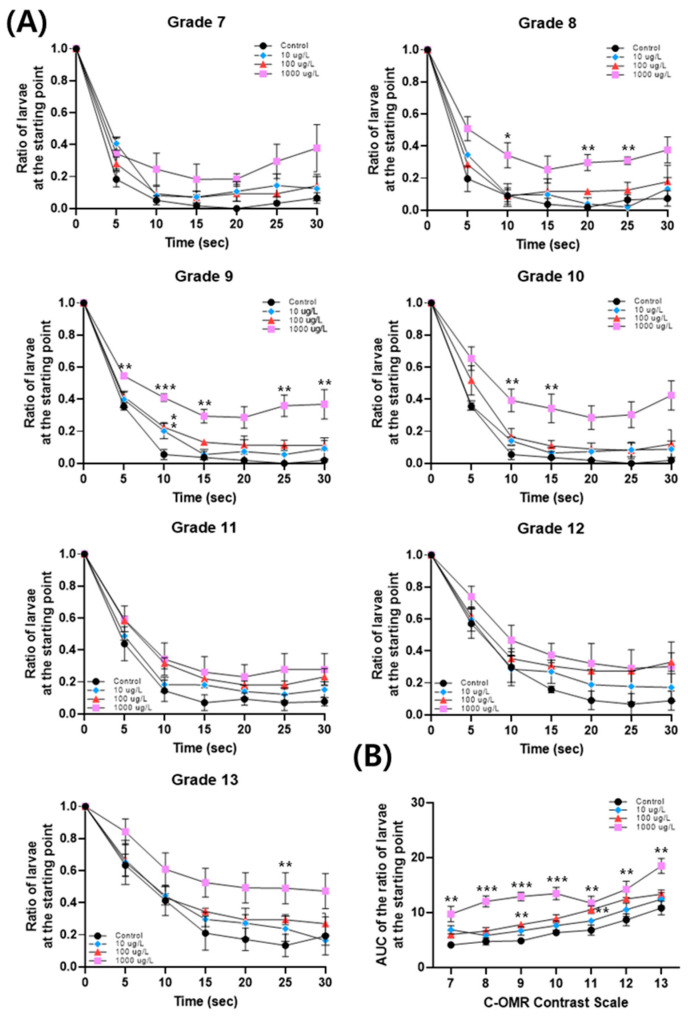
(**A**) The ratio of larvae at the starting point curve after exposure to PFOS at 10, 100, and 1000 μg/L. (**B**) The area under the curve (AUC) of the ratio of larvae at the starting point after exposure to PFOS at 10, 100, and 1000 μg/L. Significance was denoted by * *p* < 0.05, ** *p* < 0.01, and *** *p* < 0.001.

As shown in Figure 2A, the number of larvae at the starting point gradually decreased over time in all contrast sensitivity grades (G7–G13) at all PFOS concentrations tested, indicating that the zebrafish larvae responded appropriately to the band flow stimulus, and the experiment proceeded normally. For all band grades of 7–13 tested, we observed that the speed of larvae moving from the starting to the end point slowed down with PFOS concentrations. In particular, a significant difference was observed in the percentage of larvae that reached the end point from the starting point at 10 s at all concentrations in grade 9, confirming that visual behavioral abnormalities occurred at all concentrations. At the highest concentration of 1000 μg/L, significant differences were observed in grades 8, 9, 10, and 13. As shown in Figure 2B, the AUC increased at all concentrations and in all grades. Notably, at the 1000 μg/L concentration, significant differences were evident in all grades, while at lower concentrations, two significant differences were observed at 100 μg/L, and no significant differences at 10 μg/L. This indicates the PFOS concentration-dependent occurrence of visual behavioral abnormalities.

Significant changes were observed in grades 9 and 11 at 100 μg/L PFOS, with no marked differences in other bands (Figure 2B). Performing the OMR test using only a single grade 10 would have made it difficult to establish a significant correlation between PFOS exposure and ocular toxicity. Therefore, observing the visuomotor response at multiple grades, that is, employing the C-OMR test method, may be more sensitive and reliable for detecting visual behavior changes due to chemical exposure than conventionally used OMR. The C-OMR analysis was developed to investigate zebrafish visual function by applying contrast sensitivity tests to the OMR analysis stepwise, providing more extensive and accurate information about the severity and type of ocular toxicity than OMR alone [37,38]. Overall, the C-OMR assay clearly demonstrates that exposure to PFOS can impair visual function in zebrafish. Previous studies utilized OMR and OKR to assess optomotor changes in zebrafish larvae following chemical exposure, such as triphenyl phosphate and bisphenol S [36,39]. A previous study employed C-OMR for digoxin, a drug used for many cardiac conditions with color vision impairment as a side effect [23]. This study is the first to employ C-OMR for ocular toxicity assessment by chemical exposure, especially PFAS. Further studies will follow to investigate the potential of various chemicals to induce ocular toxicity by applying this C-OMR assay.

### 3.4. PFOS-Induced Altered Gene Expression

**Figure 3:**
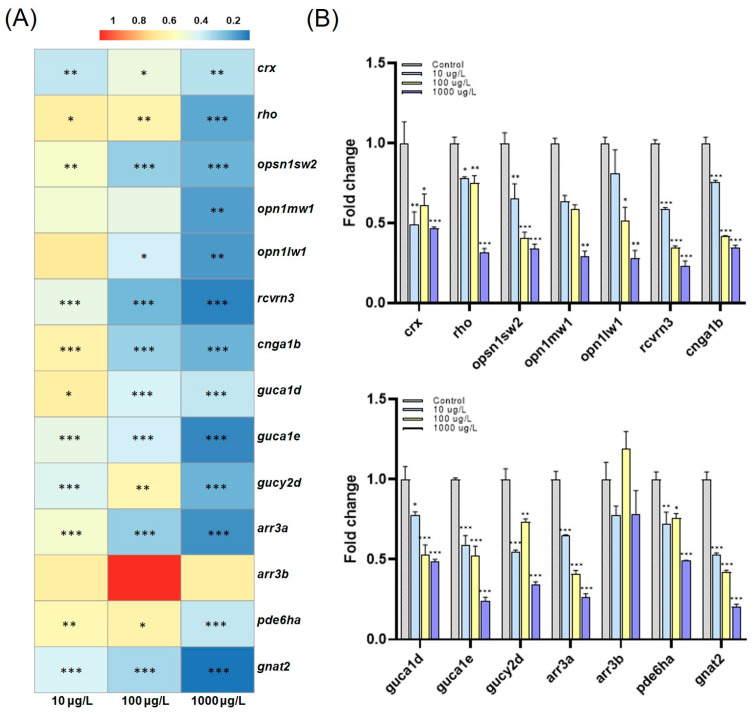
(**A**) Hierarchical clustering analysis of qPCR data. Statistical significance was denoted as * *p* < 0.05, ** *p* < 0.01, and *** *p* < 0.001. (**B**) Fold change to expression levels of *crx*, *rho*, *opsn1sw2*, *opn1mw1*, *opn1lw1*, *rcvrn3*, *cnga1b*, *guca1d*, *guca1e, gucy2d*, *arr3a*, *arr3b*, *pde6ha*, and *gnat2* in zebrafish larvae exposed to PFOS at 10, 100, and 1000 μg/L. Significance was denoted by * *p* < 0.05, ** *p* < 0.01, and *** *p* < 0.001.

To investigate the underlying mechanism of behavioral changes, we profiled expression changes in genes associated with photoreceptors and phototransduction in the retina, namely visual system disorders. As a result, we observed a significant reduction in most tested genes such as *crx*, *rho*, *opsn1sw2*, *opn1mw1*, *opn1lw1*, *rcvrn3, cnga1b*, *guca1d*, *guca1e*, *gucy2d*, *arr3a*, *gnat2*, and *pde6ha* (Figure 3). Exposure to PFOS is believed to have affected most visual function-related pathways. Studies exploring the effects of PFOS exposure on zebrafish behavior abound [40,41,42,43,44], with only a few studies reporting a causal link to visual toxicity [6,14,45]. We thus speculate that the abnormal development of pathways in photoreceptors and phototransduction, observed as the significant repression of associated gene expression, contributed to previously reported neurotoxicity and neurobehavioral changes. 

Figure 3 shows a significant concentration-dependent downregulation in photoreceptor-related genes, including *crx*, *rho*, *opsn1sw2*, *opn1mw1*, and *opn1lw1*. The downregulation of opsin genes is a potential factor in retinitis pigmentosa, which induces the degeneration of photoreceptors and eventually leads to visual impairment and blindness [46]. Although it is a human outcome, retinitis pigmentosa represents the most common form of the inherited photoreceptor degeneration resulting from degenerated rod photoreceptors [47]. The photoreceptor, a sensory organ, is responsible for converting external light stimuli into nerve signals, which subsequently travel through the nerves to the brain, resulting in alterations in cognitive processes [48]. The repressed expression of opsin genes, as found in both *rho* in rod cells and opsin gene families (*opsn1sw2*, *opn1mw1* and *opn1lw1*) in cone cells, is accompanied by decreased *crx* gene expression, which is essential for photoreceptor cell development [49]. Consequently, in the gene expression analysis, we showed that downregulated photoreceptor-related genes are linked to impaired visual behaviors in zebrafish larvae in response to PFOS exposure. 

Hertz et al. (2021) reported that abnormal expression of homeobox genes, including *crx*, whose repression by PFOS exposure as described in the previous paragraph, is accompanied by deregulated phototransduction-related genes [50]. The same result was also observed in our study, where the expression levels of phototransduction cascade-associated genes (*rcvrn3*, *guca1d*, *gucy2d*, and *guca1e*, *gnat2*, *pde6ha*, *cnga1b*, and *arr3a*) were significantly repressed concentration-dependently. A significant downregulation in the arrestin-related gene *arr3a* was also observed. The protein arrestin functions in blocking and recovering the phototransduction cascade [51], and the dysregulation of arrestins causes an abnormal influx of Ca^2+^ ions inhibited by the neurotransmitter glutamate [52]. The disrupted Ca^2+^ balance induced by these fluxes constitutes the main mechanism by which PFOS triggers neurotoxicity [53]. We thus speculate that a decrease in *arr3a* levels may mediate behavioral disorders by inducing the Ca^2+^ imbalance. The dysregulation of phototransduction-related genes has been linked to various visual disorders, including impairments in light adaptation and the coordination of visual responses [54]. Iqubal et al. (2020) reported that knocking down the phototransduction gene, *gucy2d*, causes behavior changes in zebrafish in OMR [26], and Chen et al. (2018) reported that another phototransduction gene mutant (*nbk^s342^*) leads to behavioral abnormalities in both OKR and OMR in zebrafish [55]. Collectively, downregulated genes involved in the phototransduction cascade and photoreceptor pathways contribute to abnormal visual behaviors caused by PFOS exposure. 

In conclusion, through visual behavior assays encompassing phototactic and contrast optomotor responses, we demonstrated that the legacy PFOS and PFOA caused ocular toxicity in zebrafish, which was not observed in their corresponding alternatives, PFBS and PFBA. The observed abnormal visual behaviors in PFOS-exposed zebrafish resulted from the disrupted expression of most genes involved in pathways of photoreceptors and the phototransduction cascade. A previous epidemiologic study has recently reported that exposure levels of PFAS alternatives, such as PFBA, were associated with eye-related disease incidence, as well as PFOS and PFOA [45]. This difference may stem from variations in the human and zebrafish eye structure. For example, zebrafish lack a macula, so different types of eye diseases can be observed [56]. Further research is needed to evaluate the ocular toxicity of PFAS and to obtain experimental evidence to support PFAS-induced eye diseases. We believe that this study provides novel insights into comparing and determining the potential ocular toxicity of legacy and alternative PFAS. 

## Data Availability

The data presented in this study are openly available.

## Supplementary Materials

The following supporting information can be downloaded at: https://www.mdpi.com/article/10.3390/toxics11121021/s1, Figure S1: Lethality and malformation of larval zebrafish (*n* = 32); Figure S2: Phototactic response of zebrafish larvae exposed to Gentamicin at 30 μM. Significance was denoted by * *p* < 0.05; Figure S3: The ratio of larvae at the starting point curve after exposure to Gentamicin at 30 μM. Significance was denoted by * *p* < 0.05, ** *p* < 0.01, and *** *p* < 0.001; Figure S4: The area under the curve (AUC) of the ratio of larvae at the starting point curve after exposure to Gentamicin at 30 µM. Significance was denoted by * *p* < 0.05, ** *p* < 0.01, and *** *p* < 0.001; Figure S5: (A) Illustration of the swimming tray with six swimming lanes for zebrafish larvae with video recording system. (B) Graded color classes. (C) Order of playback on a tablet in the G4, G7–G13 gray band for contrast-optomotor response assay; Table S1: The primers of qRT-PCR.
