# Supplementary material for: Comparing Ocular Toxicity of Legacy and Alternative Per- and Polyfluoroalkyl Substances in Zebrafish Larvae
Source: Toxics. 2023 Dec 14;11(12):1021. doi: 10.3390/toxics11121021 (PMC10747198; doi:10.3390/toxics11121021)
Supplement: Supplementary file 1 [file toxics-11-01021-s001.zip › toxics-2731273-supplementary.pdf]

Supplementary Materials: Comparing Ocular Toxicity of Legacy and Alternative Per- and Polyfluoroalkyl Substances in Zebrafish Larvae

Han-seul Lee, Soogyeong Jang, Youngsub Eom and Ki-Tae Kim

Table S1. The primers of qRT-PCR.

Gene	Accession ID	Sequence (5' - 3')
beta-actin	NM_181601.5	F: CGAGCTGTCTTCCCATCCA<br>R: TCACCAACGTAGCTGTCTTTCTG
rho	NM_131084	F: TCACAGTCCTGCCCAGACAT<br>R: AGAATGCCGGTCCCTCTGTA
opsn1sw2	NM_131192	F: TCACAGTCCTGCCCAGACAT<br>R: GAATACGCCACTGTGTCCCA
opn1mw1	NM_131253	F: ACTGCTCACTTGTGGGCTAC<br>R: ACCTGACCTCCAAGTGTTGC
opn1lw1	NM_001313715.1	F: GCCGAGAAGGAAGTGTCCAGAATG<br>R: AAGCAGGCGAAGAACGTGTAAGG
rcvrn3	NM_200825.2	F: GTCCTACAGGTCGCATCACA<br>R: AAACGTGTTGGGCGTAAGTG
arr3a	NM_001002405.1	F: CCTCATGGTATCAGGCGGAG<br>R: AGTCTTTGGGTTTGGGGTGC
arr3b	NM_200792.1	F: CCACGTGGAAAGCGTTGATT<br>R: CTCGTCCATAGCGAAAGGCA
crx	NM_152940.1	F: GGTCAGCCCTCTTCCTACAG<br>R: CATGGGAGAAAGGTACGGGC
gnat2	NM_131869.2	F: CTGGTGAAGCTGCCACAGTA<br>R: GCACACTTGACTGCTTGCTG
pde6ha	NM_001305554	F: GACCACTCGCACCTTCAAGA<br>R: ACAGTGATGTCTGTGCCGAG
cnga1b	XM_695944.9	F: TGTACCCGGACCCTTCAGAT<br>R: CGATGGTAGTGAGCGTCAGG
guca1d	NM_001011661.1	F: TCCGTCAGGCCTCATCACT<br>R: AAACGTGTTGGGCGTAAGTG
guca1e	NM_200656.1	F: CCTCATGGTATCAGGCGGAG<br>R: CTGGAGGATTTTGTGCGTTCA
gucy2d	NM_131866.1	F: CATCAGCTGAAGAGGTTTCTCC<br>R: ATACCCATCGCCCACCACTC

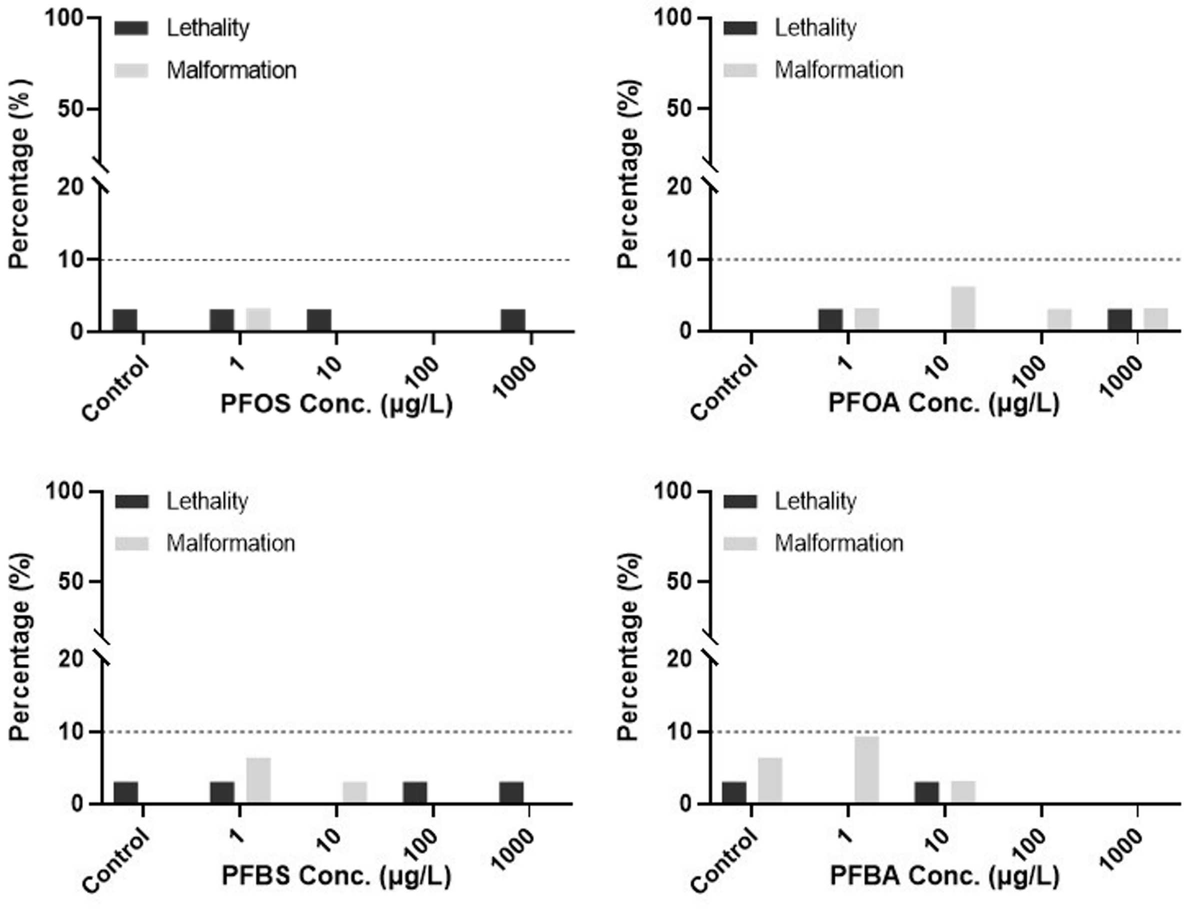

Figure S1. Lethality and malformation of larval zebrafish (n = 32).

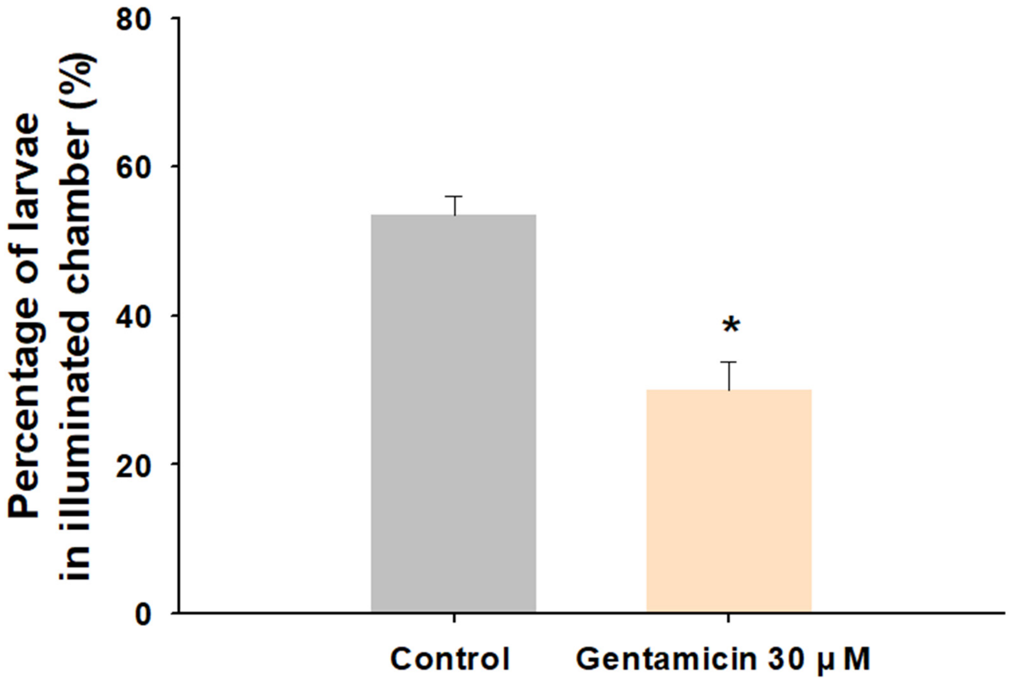

Figure S2. Phototactic response of zebrafish larvae exposed to Gentamicin at 30 μM. Significance was denoted by $^*p < 0.05$.

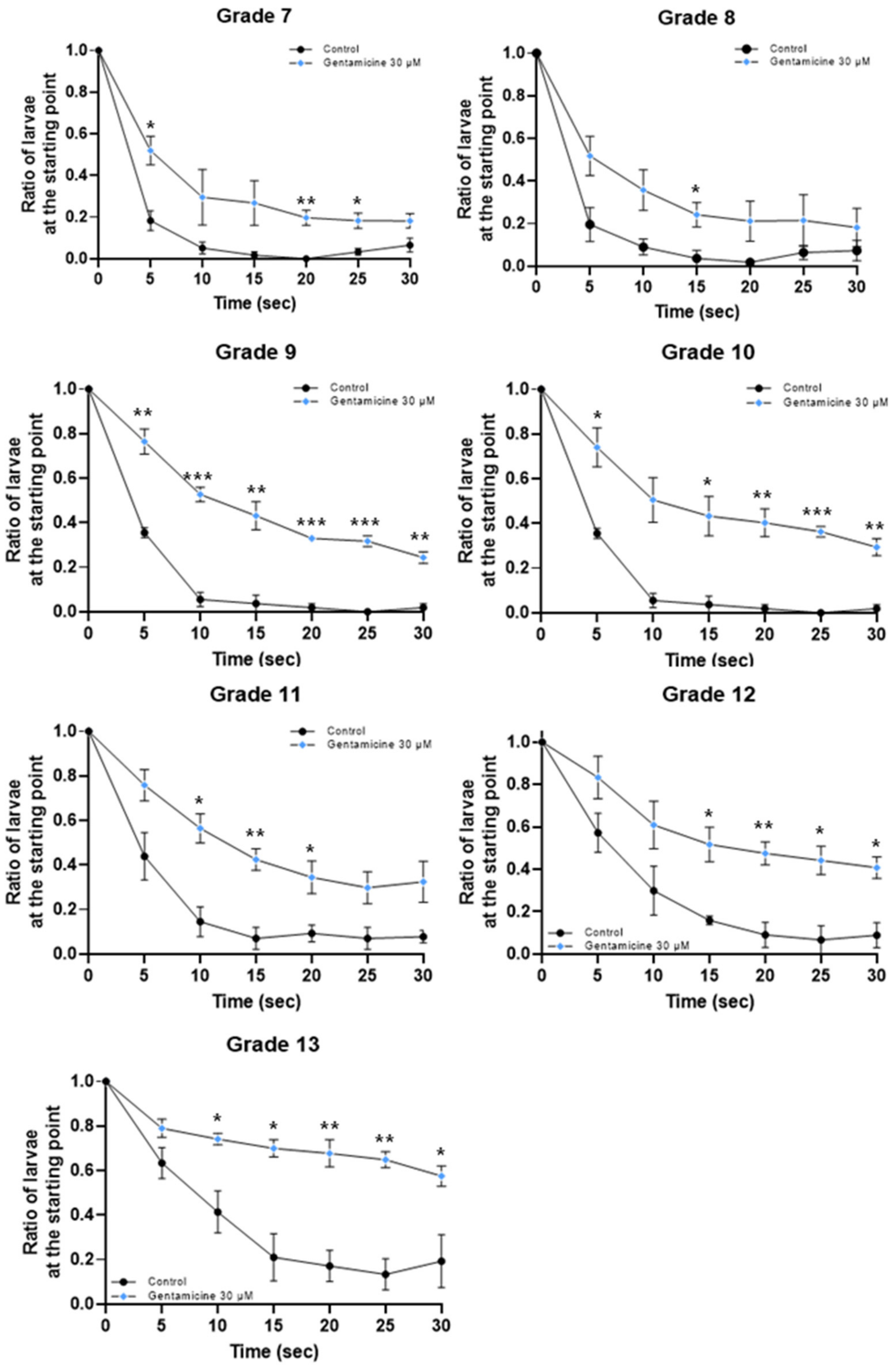

Figure S3. The ratio of larvae at the starting point curve after exposure to Gentamicin at 30 μM. Significance was denoted by **p* < 0.05, ***p* < 0.01, and ****p* < 0.001.

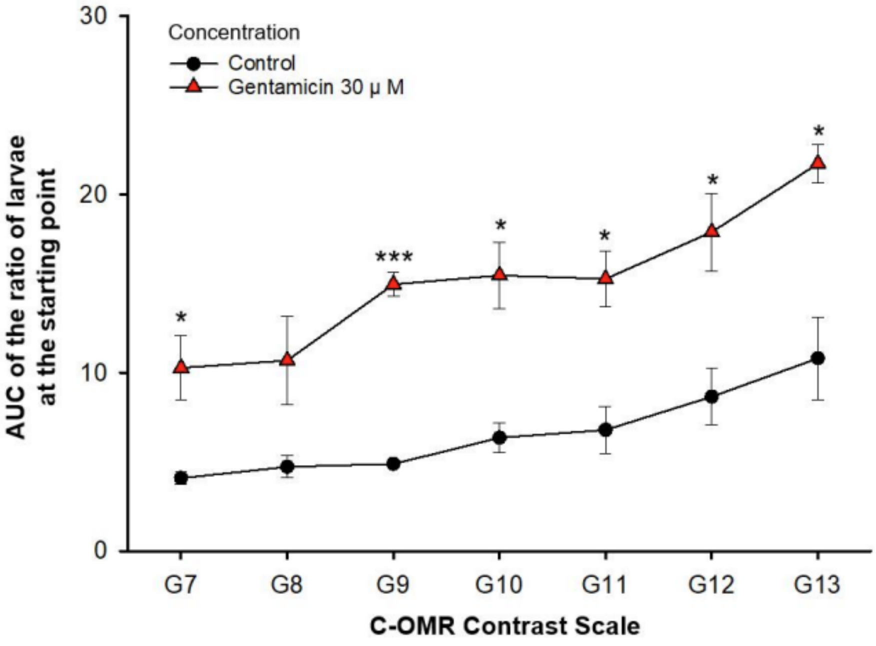

Figure S4. The area under the curve (AUC) of the ratio of larvae at the starting point curve after exposure to Gentamicin at 30 μM. Significance was denoted by *$p < 0.05$, **$p < 0.01$, and ***$p < 0.001$.

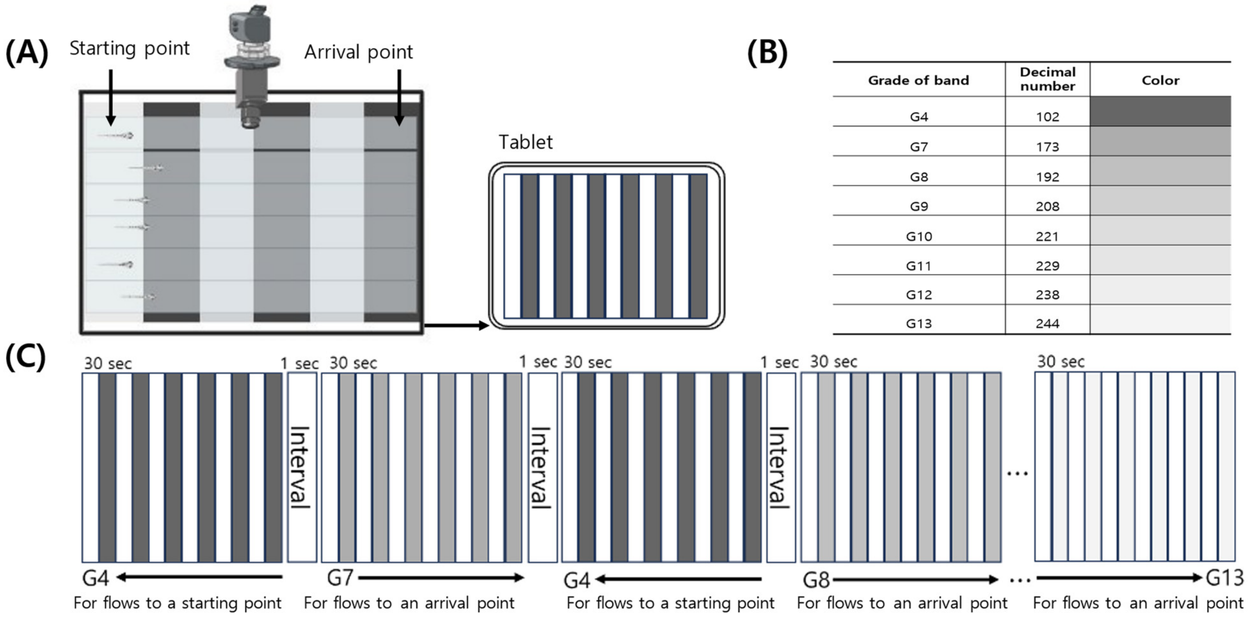

Figure S5. (A) Illustration of the swimming tray with six swimming lanes for zebrafish larvae with video recording system. **(B)** Graded color classes. **(C)** Order of playback on a tablet in the G4, G7–G13 gray band for contrast-optomotor response assay.
